# Diagnostic performance of intestinal ultrasound compared with CT enterography in Crohn’s disease: single-center experience

**DOI:** 10.3389/fmed.2025.1705629

**Published:** 2025-12-05

**Authors:** Yuanzhu Zhao, Qiong Fu, Panpan Wang, Shuru Wei, Dan Liu

**Affiliations:** ^1^Ultrasound Imaging, Heyou Hospital, Foshan, Guangdong, China; ^2^Department of Ultrasound Imaging Medicine, Zhuhai People's Hospital, Zhuhai, Guangdong, China

**Keywords:** Crohn’s disease, transabdominal bowel ultrasonography, computed tomography enterography, diagnostic efficacy, ultrasound imaging medicine

## Abstract

**Aim:**

This study aimed to clarify the diagnostic value of transabdominal bowel ultrasonography (TBUS) examination for identifying the location of Crohn’s disease (CD) lesions and their complications in relation to computed tomography enterography (CTE).

**Methods:**

A retrospective analysis of the clinical data of 100 patients diagnosed with CD in our hospital from February 2022 to December 2023 was conducted. All patients underwent regular treatment after diagnosis, and CTE and TBUS examinations were performed at a 6-month (±2 weeks) follow-up after diagnosis. The efficacy of CTE examination and TBUS examination in diagnosing CD and its complications were compared through clinical diagnosis as a final criterion, and receiver operating characteristic (ROC) curves were drawn for diagnosis.

**Results:**

The sensitivity of both diagnostic methods for intestinal wall thickening was 96.94% (95/98), indicating no statistical significance (*p* > 0.05). No statistical significance in diagnostic efficacy in diagnosing CD complicated with intestinal stenosis was exhibited between the two examination methods (*p* > 0.05). No statistical significance in diagnostic efficacy in diagnosing CD complicated with intestinal fistula was exhibited between the two examination methods (*p* > 0.05). No statistical significance in diagnostic efficacy in diagnosing CD complicated with abdominal abscess was exhibited between the two examination methods (*p* > 0.05).

**Conclusion:**

TBUS examination can achieve diagnostic results comparable to CTE, clearly displaying lesion site and intestinal segment structure of CD patients, and it exerts unique advantages in detecting extraintestinal complications. Thus, TBUS examination possesses high value in CD-assisted diagnosis and follow-up.

## Background

Crohn’s disease (CD), a chronic non-specific inflammatory granulomatous disease of the digestive tract with unknown etiology, can occur in any part of the gastrointestinal tract, involving the entire digestive tract from the mouth to the anus, and is most common in the distal small intestine and colon (1, 2). Research has demonstrated that the incidence of complications within 5 years of CD diagnosis exceeds 50%, and the incidence of complications is very high within 20 years, ranging from 70 to 94% (3). Clinical practice has validated that early diagnosis and standardized treatment are beneficial for disease control and ameliorate the long-term prognosis of CD patients (4, 5).

Endoscopic examinations, such as gastroscopy, colonoscopy, and enteroscopy, are vital diagnostic procedures for CD, although, as invasive examinations, they can enhance patients’ suffering and also pose an elevated risk of complications (6). Computed tomography enterography (CTE), one of the standard imaging methods for evaluating small intestinal enteritis lesions, combines the advantages of CT and X-ray small intestinal barium contrast imaging, which can more intuitively and clearly display changes in the diseased intestine and extraluminal space; moreover, the operation is simple, and patients’ tolerance is good (7, 8). Nevertheless, CTE is limited by radiation exposure and dose accumulation, especially for children who suffer from CD during childhood, and the radiation damage caused by long-term CT follow-up cannot be ignored (9). Currently, research has taken transabdominal bowel ultrasonography (TBUS) as a technique for diagnosing and monitoring CD intestinal lesions; its accuracy is comparable to CTE, and it exerts advantages such as economy, flexibility, and non-invasiveness (10–12). Nevertheless, there are few research reports on this technology.

Based on this, this research aimed to clarify the diagnostic value of TBUS examination for the location of CD lesions and their complications relative to CTE.

## Materials and methods

### General data

This single-center retrospective analysis on clinical data of patients diagnosed with CD in the Department of Ultrasound Imaging Medicine, Zhuhai People’s Hospital, was conducted from February 2022 to December 2023. Inclusion criteria were as follows: (1) Suspected CD due to varying degrees of diarrhea, abdominal pain, melena, and emaciation, and diagnosed with CD through endoscopic examinations, imaging examinations, histopathological examinations (such as surgical pathology), and follow-up results; (2) complete CTE and TBUS data available; and (3) age ≥18 years old. Exclusion criteria: (1) Complicated with other inflammatory bowel diseases besides CD, such as ulcerative colitis, infectious colitis, enterophthisis, intestinal tumors, etc.; (2) complicated with other autoimmune diseases; (3) those who are admitted to our hospital due to conditions requiring emergency surgical treatment for current complications (such as perianal abscess, fistula, stenosis, perforation, etc.); (4) those who were allergic to iodine and scopolamine; (5) complicated with severe heart, liver, kidney, and brain diseases; (6) those with abnormal hematopoietic or coagulation functions; and (7) pregnant women. According to the above criteria, a total of 100 CD patients were included. Among them, there were 64 men and 36 women, aged (34.16 ± 12.59) years old, and 26 cases had a history of intestinal resection surgery for CD (postoperative patients). This research obtained approval from the Medical Ethics Committee of our hospital, and all research subjects signed informed consent.

### Examination methods

All patients underwent regular treatment after diagnosis, such as aminosalicylic acid preparations, glucocorticoids, immunosuppressants, antibiotics, biologics, etc. Both CTE and TBUS examinations were conducted during the follow-up period of 6 months (±2 weeks) after diagnosis. For the vast majority of patients (92/100), both examinations were completed on the same day; for the remaining eight patients, the average interval between the two examinations was 2.5 days (range: 1–7 days). All examinations were completed before the reference standard assessment.

CTE examination: (1) the patients should fast for 8 h, and 1 day before the examination, a residue-free diet is recommended for patients. Before examination, patients should be instructed to take 2,000 mL of 2.5% isotonic mannitol solution orally and drink it multiple times within 60 min. The SIEMENS SOMATOM Definition Flash Photon Dual Source CT128 scanner should receive an application for CTE examination. During examination, patients should be placed in a supine position and scanned from the diaphragm to the lower edge of the pubic bone. Scanning parameters were as follows: tube voltage of 120 kV, tube current of 300–350 mA, matrix of 512 × 512, pitch of 0.914, and scanning layer thickness of 1.0 mm. During enhanced scanning, a non-ionic contrast agent (iohexol injection) was injected through the elbow vein with a high-pressure syringe at a speed of 2.5–3 mL/s, a contrast concentration of 100 mL/30 g, and an injection volume of 1.5–2.0 mL/kg. After injecting the contrast agent, an automatic tracking technology should be applied for dual-phase scanning of arterial and venous phases. (2) The inspection result images should be uploaded to the Siemens processing workstation, and the thin-layer image should be reconstructed after 3D maximum density projection. The axial, sagittal, and coronal planes should be observed, and the angle should be adjusted, if necessary. CTE images should be diagnosed by two radiologists with 5 years or more of experience. Both radiologists remained blinded to the clinical diagnosis of the patients and the TBUS results in this study. The site of the lesion, intestinal wall thickness, enhancement, intestinal morphology, and extraintestinal abnormalities should be observed, such as complications like fistula and abscess, as well as mesenteric lymph nodes and fat creeping. (3) Grouping should receive conduction according to Cole’s small intestine grouping method: Group 1, duodenum; Group 2, upper segment of the jejunum; Group 3, lower segment of the jejunum; Group 4, upper segment of the ileum; Group 5, middle segment of the ileum; and Group 6, lower segment of the ileum. (4) Typical CTE signs of CD during active period: significant thickening of intestinal wall (>4 mm), significant enhancement of intestinal mucosa accompanied by intestinal wall stratification, significant enhancement of mucosal inner ring and serosal outer ring, presenting a “target sign” of elevated, dilated, and twisted mesenteric vascular shadows (comb tooth sign); and corresponding increase in mesenteric fat density and blurring, as well as enlargement of mesenteric lymph nodes.

TBUS examination: The prospective TBUS examination was performed by an ultrasound expert with 5 years of experience in intestinal ultrasound. During the examination, the operator was aware of the patient’s CD diagnosis and may have had access to the patient’s previous imaging data (which is common in routine clinical follow-ups), but remained blinded to the CTE results of this follow-up. To assess inter-observer consistency, all static and dynamic images of TBUS were saved for subsequent blinded analysis. The patients should fast for 8 h, and 1 day before the examination, a residue-free diet is recommended for patients. Before examination, patients should be instructed to take 2,000 mL of 2.5% isotonic mannitol solution orally and drink it multiple times within 60 min. The SuperSonic Aixplore color Doppler ultrasound diagnostic instrument should receive application for TBUS examination, with a 3.5–5.0 MHz abdominal probe and an 8–14 MHz linear array probe. The patients should be on an empty stomach for over 8 h before examination and be placed in a supine position. During the examination, the operator systematically collects data based on a predefined structured checklist, which covers the thickness and layer structure of the intestinal wall, perivascular conditions (comb sign), and the presence of complications such as stenosis, fistula, and abscess. This aims to minimize operator-related errors and ensure standardization of the examination process. An abdominal probe (at a depth of 6–8 cm) should be applied to observe the end of the ileum, ileocecal junction, right lower quadrant abdominal small intestine, right upper quadrant abdominal small intestine, left lower quadrant abdominal small intestine, and left upper quadrant abdominal small intestine in a clockwise direction along right lower quadrant abdomen, and observe the abdominal cavity and record condition of the intestinal wall and abdominal cavity. After replacement with a linear array probe, the condition of each intestinal tube should be explored again in the above order, with a focus on observing the area of interest. The condition of the affected intestine (location, intestinal wall thickness, echo, hierarchical structure, etc.) should be observed and recorded in detail. Simultaneously, extraintestinal conditions should be observed and recorded, such as stenosis (location and number), intestinal fistula (site and number), abscess (number, site, and size), mesenteric lymph node size, and abdominal fluid accumulation (maximum anterior–posterior diameter).

### Inter-observer consistency analysis

To evaluate the reliability of TBUS diagnosis, we conducted a retrospective inter-observer consistency analysis. We invited another senior ultrasound physician (with 8 years of experience in abdominal ultrasound, including 3 years of specialization in inflammatory bowel disease ultrasound) who was unaware of the patients’ clinical information, initial TBUS reports, and CTE results, to serve as an independent reviewer. The reviewer used a standardized data collection checklist to review all 100 anonymized TBUS image datasets offline. This design ensured that the inter-observer consistency analysis was conducted under complete blindness.

### Reference standard and blinded adjudication

This study comprehensively applied the following reference standards to ensure the level of evidence: for intestinal stenosis and intestinal fistula, the final diagnosis was based on endoscopic examination (such as balloon-assisted enteroscopy) and/or findings during surgical exploration, as well as histopathological results, as the gold standard. It is important to note that in this study, the diagnosis of intestinal stenosis was based solely on morphological criteria (bowel wall thickening and luminal narrowing). The conventional TBUS protocol used did not aim to, and cannot, reliably differentiate between inflammatory and fibrotic stenosis. For abdominal abscess, the final diagnosis was based on the culture of the puncture drainage fluid under imaging (CT or ultrasound) guidance, or findings during surgical exploration, as the gold standard. All examinations for the above reference standards were completed within ±2 weeks after the follow-up examinations of TBUS and CTE.

To ensure the objectivity of the reference standard adjudication, a multidisciplinary team consisting of at least two gastroenterology experts, one radiology expert, and one surgery expert reviewed the clinical, endoscopic, pathological, and radiological drainage/surgical records of all patients without any knowledge of the TBUS and CTE results in this study and ultimately adjudicated whether there were complications of interest in this study. This process minimized inclusion bias.

### Comparison of imaging diagnosis with reference standards

The imaging diagnosis of TBUS and CTE was based on the aforementioned imaging characteristics. Intestinal fistula was defined as a hypoechoic tract extending from the bowel lumen through the wall to another bowel loop, an adjacent organ, or the skin. The presence of gas bubbles (manifesting as moving bright echoes) or fluid within the tract provided additional supportive evidence. Abscess was defined as a localized, predominantly hypoechoic or anechoic collection with irregular margins, with or without internal gas echoes. A lesion with the largest diameter of >2 cm was considered diagnostic. TBUS was defined as a wall thickness ≥4 mm, often accompanied by loss of the normal stratified wall structure, decreased echogenicity of the submucosa, and hypervascularity on color Doppler imaging. Subsequently, these imaging diagnosis results are compared with the reference standards defined above and adjudicated by a blinded multidisciplinary team to calculate diagnostic performance indicators. Specifically, intestinal stenosis: The imaging diagnosis is compared with the stenosis and its pathological results observed during endoscopy/surgery. Intestinal fistula: The imaging diagnosis is compared with the fistula and its pathological results observed during endoscopy/surgery. Abdominal abscess: The imaging diagnosis is compared with the pus observed during puncture, + drainage/surgery. Intestinal wall thickening: As a basic imaging indicator, it is confirmed by integrating results from endoscopy, other imaging modalities, and clinical follow-up.

### Statistical analysis

The evaluation of diagnostic performance is based on the original diagnostic results (such as presence/absence) rather than a predetermined single diagnostic threshold. We calculated the sensitivity, specificity, accuracy, and the corresponding 95% confidence intervals (CIs) for each complication diagnosed by CTE and TBUS. Simultaneously, receiver operating characteristic (ROC) curves were plotted, and the areas under the curves and their 95% CIs were calculated. The DeLong test was used to statistically compare the AUCs of TBUS and CTE to assess the differences in diagnostic performance between the two methods. A *p* value < 0.05 was considered statistically significant. All statistical analyses were performed using SPSS 27.0 and R software (version 4.2.1, using the pROC package for the DeLong test).

## Results

### Inter-observer consistency

Regarding the consistency analysis between the prospective TBUS operators and independent blinded readers, Cohen’s kappa values indicated excellent inter-observer agreement in the diagnosis of intestinal wall thickening (*κ* = 0.91), intestinal stenosis (*κ* = 0.88), intestinal fistula (*κ* = 0.85), and abdominal abscess (*κ* = 0.89) (all *p* < 0.001). This suggests a high degree of reliability in TBUS diagnosis among different operators.

### Data analysis of patient characteristics and examination results

At the time of the 6-month follow-up imaging, all patients were on regular medical therapy for CD maintenance. The treatment profile of the cohort was as follows: 58% (58/100) of patients were on biologic agents (e.g., anti-TNFα, anti-integrins), 35% (35/100) were on immunomodulators (azathioprine/6-mercaptopurine or methotrexate), 25% (25/100) were on 5-aminosalicylates, and 18% (18/100) were receiving oral corticosteroids, typically in a tapering regimen. A considerable number of patients were on combination therapy, most commonly a biologic agent combined with an immunomodulator. This treatment mix reflects real-world clinical practice for moderate to severe CD in the maintenance phase.

Among 100 CD patients, two patients’ colonoscopy results depicted healing of the intestinal wall mucosa, and the TBUS and CTE tests were negative due to regular treatment. Among the 98 patients included in the analysis, the diagnosis of all complications (42 cases of stenosis, 25 cases of fistula, and 15 cases of abscess) was ultimately confirmed by a blinded multidisciplinary team based on reference standards such as endoscopy, pathology, or puncture/surgery. Among the remaining 98 CD patients, TBUS examination revealed intestinal wall thickening in 95 cases, and CTE examination revealed intestinal wall thickening in 95 cases. The sensitivity of both diagnostic methods for intestinal wall thickening was 96.94% (95/98), indicating no statistical significance (*p* > 0.05; Table 1).

**Table 1:** TBUS and CTE examination results of CD patients.

Examination method	Bowel wall thickening [*n* (%)]	Inter-observer agreement (Kappa) for TBUS
TBUS	95 (96.94)	0.91
CTE	95 (96.94)	–
*χ* ^2^	0	
*P* value	1	

### Diagnostic efficacy of TBUS or CTE in diagnosing CD complicated with intestinal stenosis

**Figure 1:**
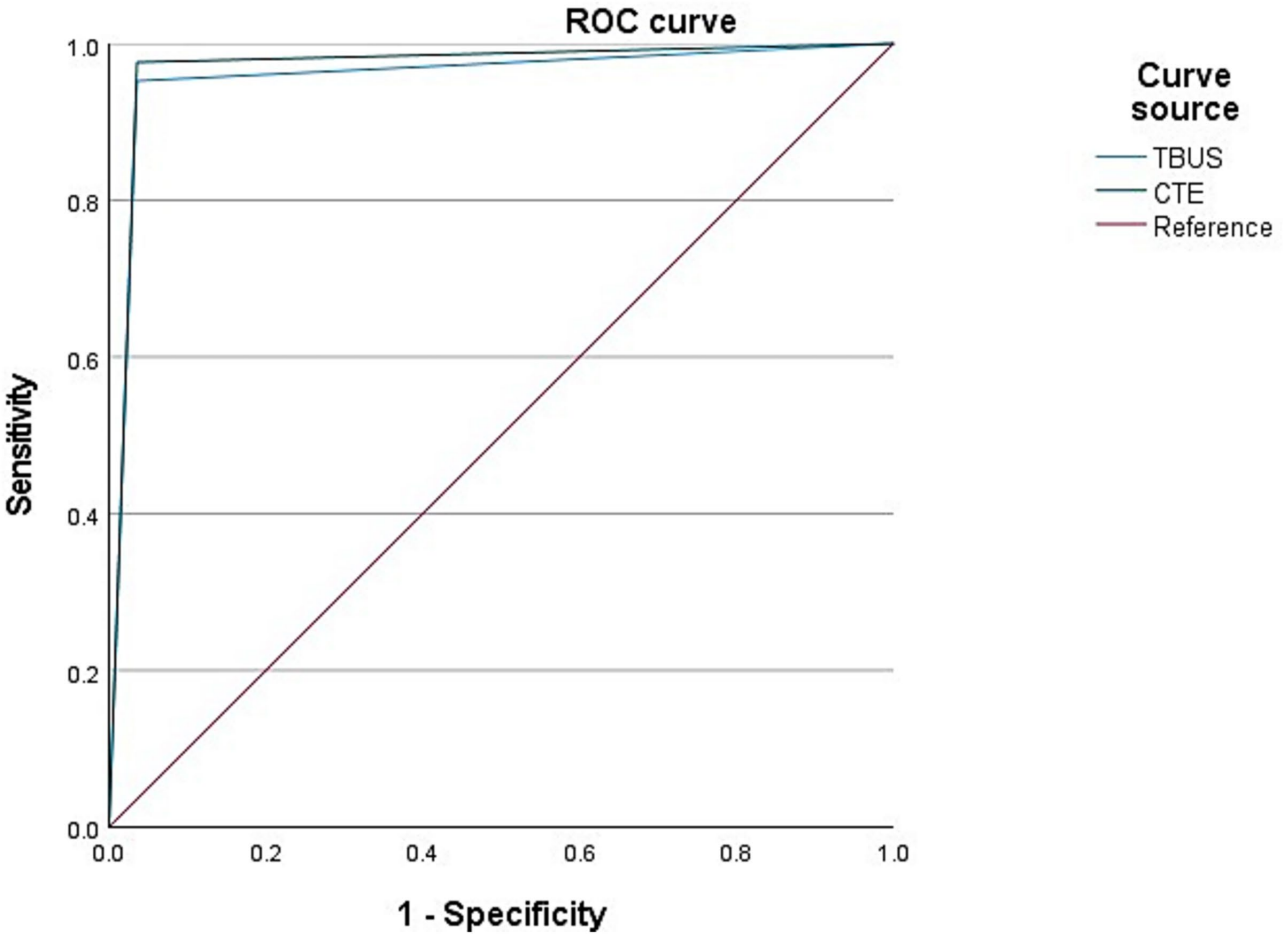
Receiver operating characteristic (ROC) curves for diagnosing intestinal stenosis. The area under the curve (AUC) for transabdominal bowel ultrasonography (TBUS) was 0.958 (95% CI: 0.911–1.000). The AUC for computed tomography enterography (CTE) was 0.970 (95% CI: 0.931–1.000). The difference between the two AUCs was not statistically significant (DeLong test, *p* = 0.56).

Among 98 patients with CD, a total of 42 cases were complicated with intestinal stenosis; 42 cases were diagnosed with intestinal stenosis through TBUS, and 43 cases were diagnosed with intestinal stenosis through CTE. Based on endoscopic examination or histopathological results as the “gold standard,” sensitivity, specificity, and accuracy of TBUS in diagnosing CD complicated with intestinal stenosis were 95.24% (40/42), 96.43% (54/56), and 95.92% (94/98), respectively, while sensitivity, specificity, and accuracy of CTE in diagnosing CD complicated with intestinal stenosis were 97.62% (41/42), 96.43% (54/56), and 96.94% (95/98), respectively. There was no statistical difference in the area under the curve (AUC) for diagnosing intestinal stenosis between TBUS and CTE (DeLong test, *p* = 0.56). The AUC for diagnosing intestinal stenosis with TBUS was 0.958 (95% CI: 0.911–1.000), while the AUC for CTE was 0.970 (95% CI: 0.931–1.000) (Table 2 and Figure 1).

**Table 2:** Diagnostic performance of TBUS and CTE for intestinal stenosis.

Examination method	Sensitivity % (*n*/*N*; 95% CI)	Specificity % (*n*/*N*; 95% CI)	Accuracy % (*n*/*N*; 95% CI)	AUC (95% CI)	Inter-observer agreement (Kappa) for TBUS
TBUS	95.24 (40/42; 84.76–98.72)	96.43 (54/56; 87.92–99.07)	95.92 (94/98; 89.95–98.50)	0.958 (0.911–1.000)	0.88
CTE	97.62 (41/42; 87.74–99.87)	96.43 (54/56; 87.92–99.07)	96.94 (95/98; 91.27–99.20)	0.970 (0.931–1.000)	–
*P* value (DeLong test)				0.56	

### Diagnostic efficacy of TBUS or CTE in diagnosing CD complicated with intestinal fistula

**Figure 2:**
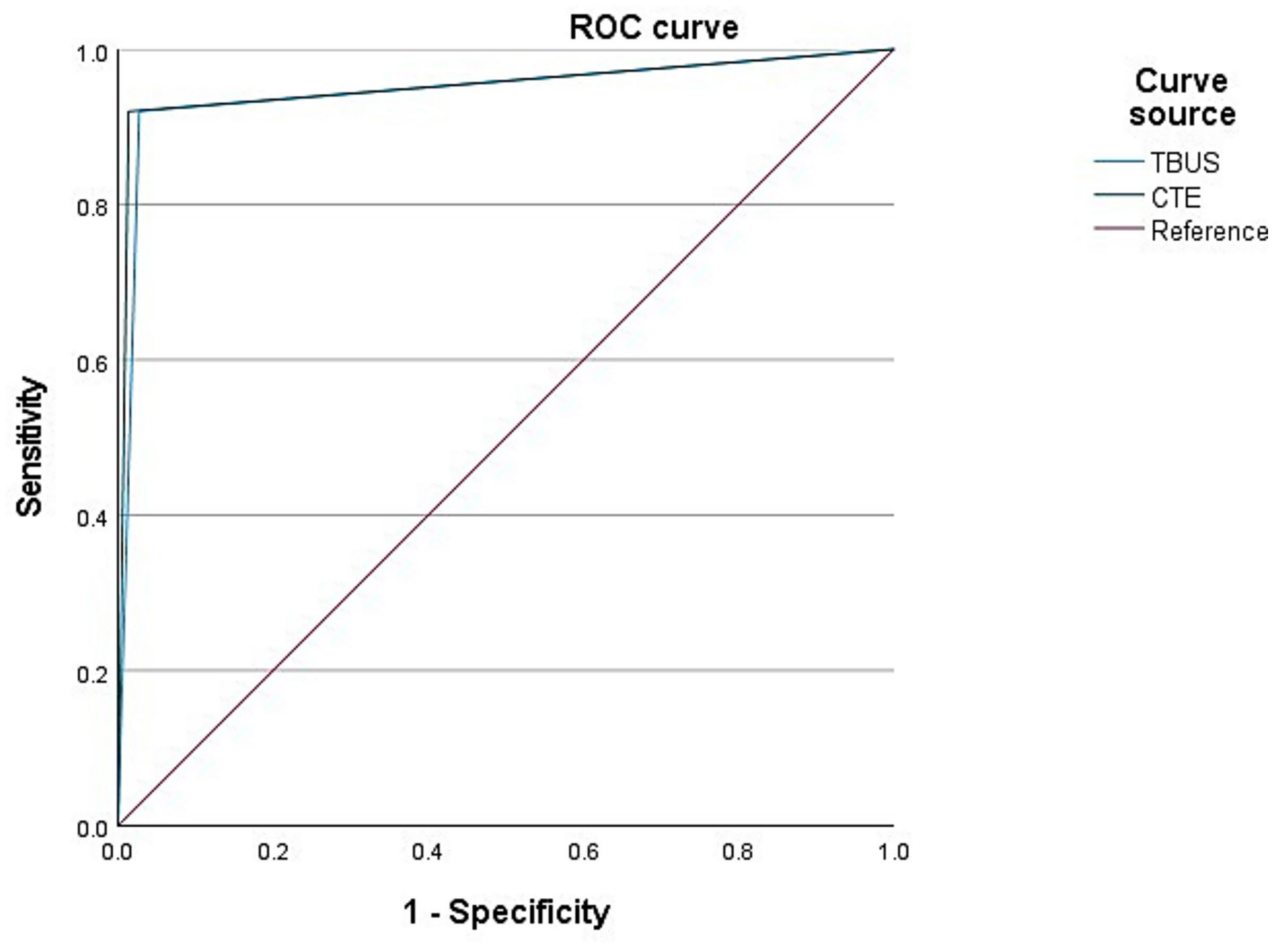
Receiver operating characteristic (ROC) curves for diagnosing intestinal fistula. The area under the curve (AUC) for transabdominal bowel ultrasonography (TBUS) was 0.946 (95% CI: 0.881–1.000). The AUC for computed tomography enterography (CTE) was 0.953 (95% CI: 0.889–1.000). The difference between the two AUCs was not statistically significant (DeLong test, *p* = 0.78).

Among 98 CD patients, a total of 25 cases were complicated with intestinal fistulas, 25 cases were diagnosed with intestinal fistula through TBUS, and 24 cases were diagnosed with intestinal fistula through CTE. Based on endoscopic examination or histopathological results as the “gold standard,” sensitivity, specificity, and accuracy of TBUS in diagnosing CD complicated with intestinal fistula were 92.00% (23/25), 97.26% (71/73), and 95.92% (94/98), respectively, while sensitivity, specificity, and accuracy of CTE in diagnosing CD complicated with intestinal fistula were 92.00% (23/25), 98.63% (72/73), and 96.94% (95/98), respectively. There was no statistical difference in diagnostic efficacy between the two methods (DeLong test, *p* = 0.78). The AUC of TBUS for diagnosing intestinal fistula was 0.946 (95% CI: 0.881–1.000), while the AUC of CTE was 0.953 (95% CI: 0.889–1.000) (Table 3 and Figure 2).

**Table 3:** Diagnostic performance of TBUS and CTE for intestinal fistula.

Examination method	Sensitivity % (*n*/*N*; 95% CI)	Specificity % (*n*/*N*; 95% CI)	Accuracy % (*n*/*N*; 95% CI)	AUC (95% CI)	Inter-observer agreement (Kappa) for TBUS
TBUS	92.00 (23/25; 75.03–97.78)	97.26 (71/73; 90.52–99.52)	95.92 (94/98; 89.95–98.50)	0.946 (0.881–1.000)	0.85
CTE	92.00 (23/25; 75.03–97.78)	98.63 (72/73; 92.70–99.96)	96.94 (95/98; 91.27–99.20)	0.953 (0.889–1.000)	–
*P* value (DeLong test)				0.78	

### Diagnostic efficacy of TBUS or CTE in diagnosing CD complicated with abdominal abscess

**Figure 3:**
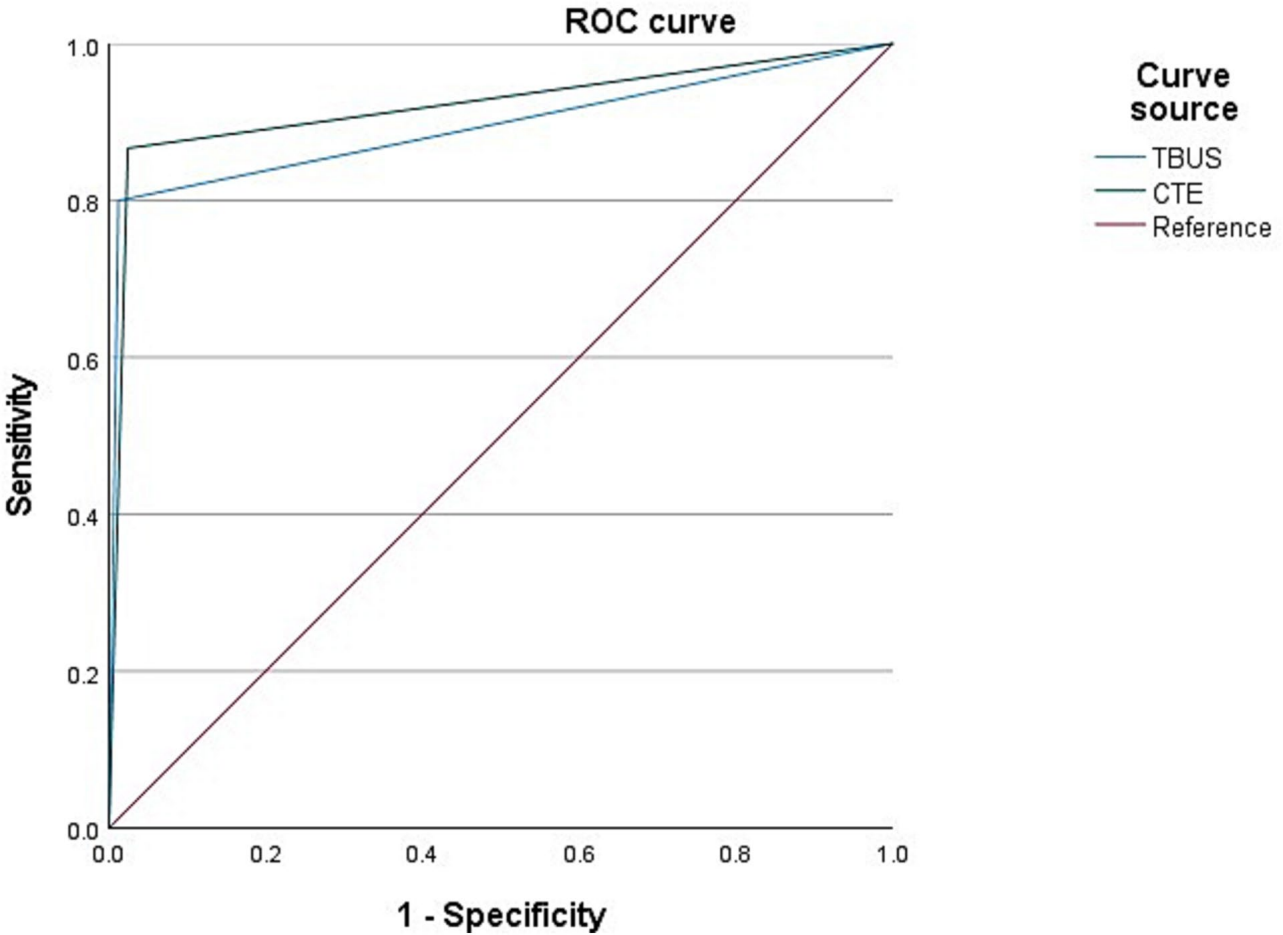
Receiver operating characteristic (ROC) curves for diagnosing abdominal abscess. The area under the curve (AUC) for transabdominal bowel ultrasonography (TBUS) was 0.894 (95% CI: 0.772–1.000). The AUC for computed tomography enterography (CTE) was 0.921 (95% CI: 0.818–1.000). The difference between the two AUCs was not statistically significant (DeLong test, *p* = 0.42).

Among 98 CD patients, a total of 15 cases were complicated with abdominal abscesses; 13 cases were diagnosed with abdominal abscesses through TBUS, and 15 cases were diagnosed with abdominal abscesses through CTE. Based on endoscopic examination or histopathological results as the “gold standard,” sensitivity, specificity, and accuracy of TBUS in diagnosing CD complicated with abdominal abscesses were 80.00% (12/15), 98.80% (82/83), and 95.92% (94/98), respectively, while sensitivity, specificity, and accuracy of CTE in diagnosing CD complicated with abdominal abscesses were 86.67% (13/15), 97.59% (81/83), and 95.92% (94/98), respectively. Although the sensitivity of TBUS is numerically lower than that of CTE (80.00% vs. 86.67%), there was no statistical difference in the AUC between the two methods (DeLong test, *p* = 0.42). The AUC of TBUS for diagnosing abdominal abscesses was 0.894 (95% CI: 0.772–1.000), while the AUC of CTE was 0.921 (95% CI: 0.818–1.000) (Table 4 and Figure 3).

**Table 4:** Diagnostic performance of TBUS and CTE for abdominal abscess.

Examination method	Sensitivity % (*n*/*N*; 95% CI)	Specificity % (*n*/*N*; 95% CI)	Accuracy % (*n*/*N*; 95% CI)	AUC (95% CI)	Inter-observer agreement (Kappa) for TBUS
TBUS	80.00 (12/15; 54.81–92.95)	98.80 (82/83; 93.66–99.94)	95.92 (94/98; 89.95–98.50)	0.894 (0.772–1.000)	0.89
CTE	86.67 (13/15; 62.12–96.26)	97.59 (81/83; 91.61–99.58)	95.92 (94/98; 89.95–98.50)	0.921 (0.818–1.000)	–
*P* value (DeLong test)				0.42	

### Subgroup analysis for diagnostic efficacy of TBUS or CTE postoperative vs. non-postoperative patients

To evaluate the impact of previous intestinal surgery on diagnostic efficacy, we conducted a subgroup analysis, dividing patients into a postoperative group (*n* = 26) and a non-postoperative group (*n* = 72). There were no significant differences in age and gender distribution between the two groups. In the postoperative group, the distribution of complications was as follows: 12 cases (46.2%) of intestinal stenosis, 8 cases (30.8%) of intestinal fistula, and 5 cases (19.2%) of abdominal abscess.

As shown in Supplementary Table S1, in both the postoperative and non-postoperative subgroups, TBUS and CTE demonstrated comparable performance in terms of sensitivity, specificity, and accuracy for the diagnosis of intestinal stenosis, intestinal fistula, and abdominal abscess. No statistical differences were observed in all inter-group comparisons (all *p* > 0.05). Specifically, for postoperative patients, the sensitivity of TBUS for diagnosing intestinal stenosis, intestinal fistula, and abdominal abscess was 91.7, 87.5, and 80.0%, respectively; for non-postoperative patients, the corresponding sensitivities were 96.7, 94.1, and 80.0%, respectively. These results indicate that the diagnostic efficacy of TBUS did not significantly decrease due to the patients’ previous surgical history.

## Discussion

CD is a chronic, inflammatory disease that can affect the entire digestive tract, and its clinical manifestations are primarily abdominal pain, diarrhea, and weight loss, often accompanied by complications, such as intestinal stenosis, fistula, and abdominal abscess (13). Research has depicted that CD can be limited to the small intestine or colon and can also involve both the small intestine and colon simultaneously (14). In CD imaging examination, CTE has always been dominant in intestinal imaging examination due to its ability to clearly observe intestinal and extraluminal lesions, simple operation, and good patient tolerance (15). A foreign report has validated that TBUS has high sensitivity and specificity for CD and has received wide attention in the diagnosis of CD (16).

The normal intestinal wall thickness is generally less than 3 mm, and its ultrasound manifestation is a well-defined five-layer structure (17). Segmental thickening of the intestinal wall is the most characteristic imaging manifestation of CD, and the thickness of the affected intestinal wall is of great significance for the diagnosis and assessment of CD activity (18). Gao et al. showed that 3–4 mm was generally regarded as a critical value for intestinal wall thickening (19). This research applied 4 mm as a critical value for intestinal wall thickening, and the results depicted that, except for two cases who did not present intestinal wall thickening due to regular treatment, 95 out of the remaining 98 cases were found to have varying degrees of intestinal wall thickening through TBUS and CTE examinations. This indicates that TBUS has a high consistency with CTE in diagnosing intestinal wall thickening.

Common complications in CD patients include intestinal stenosis, mesenteric lymph node enlargement, intestinal fistula, obstruction, and abscess formation (20). Intestinal stenosis is the most common complication of CD, and 20% of patients exhibited no obvious symptoms (21). The intestinal stenosis of CD includes inflammatory stenosis and fibrous stenosis, and inflammatory stenosis can be intervened with drug therapy, while fibrous stenosis requires surgical treatment. TBUS can display a narrowed portion of the intestine and a dilated intestine before stenosis. Herein, TBUS and CTE examinations exhibited high sensitivity, specificity, and accuracy in detecting CD complicated with intestinal stenosis. As verified by the ROC curve, both examination methods exhibited high diagnostic efficacy in detecting CD complicated with intestinal stenosis. Nevertheless, it has been depicted that conventional ultrasound has a high sensitivity and specificity for intestinal stenosis, but it cannot distinguish between inflammatory stenosis and fibrous stenosis (22). The TBUS technique employed in this study, while excellent for detection, has inherent limitations in characterizing tissue composition and thus in making this crucial distinction. This delineates a clear and important frontier for the evolution of intestinal ultrasound. Emerging advanced ultrasound techniques, specifically contrast-enhanced ultrasound (CEUS) and shear-wave elastography (SWE), offer promising solutions. Prospective studies with surgical correlation have demonstrated that CEUS can provide valuable functional information on disease activity and complicated lesions (23). CEUS allows for the quantitative assessment of microvascular perfusion, which is typically increased in inflammatory tissue, while SWE measures tissue stiffness, which is elevated in fibrotic strictures. Future studies integrating these functional and biomechanical assessments with conventional TBUS are highly warranted to create a comprehensive, non-invasive tool for guiding personalized therapeutic strategies in CD patients with stricturing disease.

Intestinal fistula is also a common complication in CD patients. 20–85% of CD patients will develop intestinal fistulas. Currently, surgery is the gold standard for diagnosing intestinal fistulas, but not every CD patient undergoes surgical management. Thus, applying surgery as the gold standard for diagnosing the presence of intestinal fistulas may have some bias. Herein, there were two cases of false negatives in CTE examination, which were terminal ileal fistulas; CTE examination only demonstrated strip exudation in this area, while TBUS examination could clearly present a low echoic strip shape. This indicates that ultrasound examination may be more effective in diagnosing fistulas than CTE, and caution should be exercised against the possibility of intestinal fistulas when linear exudates are detected in CTE. There were two cases of false negatives in TBUS examination, which were intestinal fistulas in the lower segment of the jejunum and the middle segment of the ileum. The major reason for the missed diagnosis in these two cases may be that they are located deep, and the small intestine in the abdominal cavity is complex and difficult to see. Two cases were misdiagnosed in the TBUS examination, and a sigmoid fistula was diagnosed as a small intestine fistula. This has a relation to the high mobility of the distal part of the sigmoid colon, which is greatly affected by intestinal gas and skeleton, whereas CTE is less susceptible to these two elements, and its diagnostic efficacy of intestinal lesions in this area is higher. According to the ROC curve, both TBUS and CTE examinations exhibited higher diagnostic efficacy for CD complicated with intestinal fistula. Moreover, the sensitivity of TBUS in detecting abdominal abscesses exhibited a slight depletion relative to that of CTE. The ROC curve depicted that both TBUS and CTE examinations presented higher diagnostic efficacy for CD complicated with abdominal abscess. TBUS may receive interference, such as intestinal gas, position, etc.

In addition, our subgroup analysis of 26 postoperative patients revealed that TBUS has comparable diagnostic efficacy to CTE in detecting stenosis, fistulas, and abscesses in this challenging patient population, with no significant decrease (see Supplementary Table S1). This preliminarily suggests that, despite the potential increase in imaging interpretation difficulty due to anatomical changes after surgery, ultrasonographers with standardized training can still obtain reliable results using TBUS. This finding supports the application of TBUS in the long-term follow-up of CD patients, including a considerable number of postoperative patients.

The excellent diagnostic performance and high inter-observer consistency exhibited by TBUS in this study provide support for its promotion in clinical practice. To achieve this result, operators need to undergo specialized training. Our center’s experience is that after having a basic understanding of conventional abdominal ultrasound, ultrasound physicians need to complete at least 6 months of specialized training on IBD ultrasound, with approximately 50 cases, under the guidance of experienced mentors, and maintain follow-up examinations for no less than 50 CD patients each year to maintain diagnostic proficiency. Combining the standardized checklist used in this study helps to achieve standardized image acquisition and interpretation among different operators. Our findings that TBUS demonstrates comparable diagnostic performance to CTE for complications support its use not only for diagnosis but also for disease monitoring. This is particularly relevant given the growing body of evidence, including a recent multicenter study, which confirms the utility of intestinal ultrasound in objectively assessing early treatment response to biologic therapy in CD patients (24).

In the design of this study, the operator of the prospective TBUS failed to remain blinded to clinical information and previous imaging, which theoretically introduces assessment bias. Specifically, knowledge of the patient’s CD diagnosis and medical history may make the operator more sensitive to abnormal findings during scanning and interpretation, potentially overestimating the diagnostic performance of TBUS (i.e., generating an optimistic bias). However, there are several points to consider to fully evaluate the impact of this bias: First, this setting reflects the typical application scenario of TBUS in real-world clinical practice. Second, and more importantly, our subsequent inter-observer consistency analysis, involving completely blinded independent readers, yielded excellent Kappa values (all >0.85) that were highly consistent with the prospective examination. This indicates that even with potential optimistic bias, the diagnostic value of TBUS itself is robust and reproducible. Nevertheless, the ideal research design would still be to keep all imaging evaluators blinded to all other information.

## Conclusion

TBUS examination can achieve diagnostic results comparable to CTE, clearly displaying the lesion site and intestinal segment structure of CD patients, and it exerts unique advantages in detecting extraintestinal complications. Thus, TBUS examination possesses high value in CD-assisted diagnosis and follow-up. Nevertheless, this research still has certain limitations, such as being a single-center, retrospective study with a small sample size, and not including patients receiving surgical treatment. Although the retrospective blinded analysis demonstrated excellent interobserver consistency, all prospective TBUS examinations were conducted by a single operator. While this design ensured consistency in examination techniques, it may have limited the generalizability of the study results to operators with different levels of experience or across different medical centers. Moreover, the operator did not maintain blinding to clinical information and previous imaging during the examination, which may theoretically introduce an optimism bias. A very important point is that the imaging assessments in this study were conducted at the “monitoring” phase, which is 6 months after diagnosis and when the vast majority of patients were under pharmacotherapy. We failed to systematically collect and report clinical indices of disease activity (such as CDAI) or endoscopic indices (such as SES-CD) at the time of imaging examination in all patients. Therefore, the results of this study are most representative of CD patients in the disease maintenance/remission phase or mildly active phase. Treatment (especially biologics and glucocorticoids) may alter the imaging significance of intestinal inflammation and complications, such as reducing intestinal wall thickening and edema. Hence, our conclusions should be cautiously extended to untreated patients in the acute flare-up phase or moderately to severely active phase, and the diagnostic performance of TBUS in these populations may need further evaluation. Despite our subgroup analysis of postoperative patients yielding encouraging results, the sample size in this subgroup, particularly the limited number of positive cases for certain complications, may not be sufficient to detect minor differences between the two groups. Future multicenter, prospective studies with larger sample sizes are needed to further confirm the diagnostic performance of TBUS in patients after CD surgery.

## Data Availability

The original contributions presented in the study are included in the article/Supplementary material, further inquiries can be directed to the corresponding author.
