# Supplementary material for: Diagnostic performance of intestinal ultrasound compared with CT enterography in Crohn’s disease: single-center experience
Source: Front Med (Lausanne). 2025 Dec 5;12:1705629. doi: 10.3389/fmed.2025.1705629 (PMC12714986; doi:10.3389/fmed.2025.1705629)
Supplement: Supplementary file 1 [file Table_1.docx]

Supplementary Table S1 Diagnostic Performance of TBUS and CTE in Postoperative and Non-postoperative Subgroups

Table S1A Intestinal Stenosis

| **Subgroup** | **Examination Method** | **Sensitivity % (n/N; 95% CI)** | **Specificity % (n/N; 95% CI)** | **Accuracy % (n/N; 95% CI)** | **P value for Sensitivity** |
| --- | --- | --- | --- | --- | --- |
| **Postoperative** (n=26) | TBUS | 91.7 (11/12; 64.6-98.5) | 92.9 (13/14; 68.5-98.7) | 92.3 (24/26; 75.9-97.9) | 0.54 |
|  | CTE | 100.0 (12/12; 75.8-100.0) | 92.9 (13/14; 68.5-98.7) | 96.2 (25/26; 81.1-99.3) |  |
| **Non-postoperative** (n=72) | TBUS | 96.7 (29/30; 83.3-99.4) | 97.6 (41/42; 87.7-99.6) | 97.2 (70/72; 90.6-99.2) | 0.56 |
|  | CTE | 96.7 (29/30; 83.3-99.4) | 97.6 (41/42; 87.7-99.6) | 97.2 (70/72; 90.6-99.2) |  |

Table S1B Intestinal Fistula

| **Subgroup** | **Examination Method** | **Sensitivity % (n/N; 95% CI)** | **Specificity % (n/N; 95% CI)** | **Accuracy % (n/N; 95% CI)** | **P value for Sensitivity** |
| --- | --- | --- | --- | --- | --- |
| **Postoperative** (n=26) | TBUS | 87.5 (7/8; 52.9-97.8) | 100.0 (18/18; 82.4-100.0) | 96.2 (25/26; 81.1-99.3) | 0.32 |
|  | CTE | 100.0 (8/8; 67.6-100.0) | 100.0 (18/18; 82.4-100.0) | 100.0 (26/26; 87.1-100.0) |  |
| **Non-postoperative** (n=72) | TBUS | 94.1 (16/17; 73.0-99.0) | 96.4 (53/55; 87.7-99.0) | 95.8 (69/72; 88.5-98.6) | 1.00 |
|  | CTE | 88.2 (15/17; 65.7-96.7) | 98.2 (54/55; 90.4-99.9) | 95.8 (69/72; 88.5-98.6) |  |

Table S1C Abdominal Abscess

| **Subgroup** | **Examination Method** | **Sensitivity % (n/N; 95% CI)** | **Specificity % (n/N; 95% CI)** | **Accuracy % (n/N; 95% CI)** | **P value for Sensitivity** |
| --- | --- | --- | --- | --- | --- |
| **Postoperative** (n=26) | TBUS | 80.0 (4/5; 37.6-96.4) | 100.0 (21/21; 84.5-100.0) | 96.2 (25/26; 81.1-99.3) | 1.00 |
|  | CTE | 80.0 (4/5; 37.6-96.4) | 95.2 (20/21; 77.3-99.2) | 92.3 (24/26; 75.9-97.9) |  |
| **Non-postoperative** (n=72) | TBUS | 80.0 (8/10; 49.0-94.3) | 98.4 (61/62; 91.5-99.9) | 95.8 (69/72; 88.5-98.6) | 0.16 |
|  | CTE | 90.0 (9/10; 59.6-98.2) | 98.4 (61/62; 91.5-99.9) | 97.2 (70/72; 90.6-99.2) |  |

P value for the comparison of sensitivity between TBUS and CTE within the same subgroup, calculated using McNemar's test.

Abbreviations: TBUS, transabdominal bowel ultrasonography; CTE, computed tomography enterography; CI, confidence interval.
